# Anterior mitral leaflet length and mitral annulus diameter impact the echocardiographic outcome after isolated myectomy

**DOI:** 10.1186/s13019-019-1037-1

**Published:** 2019-12-05

**Authors:** Mateusz Kuć, Magdalena Kumor, Mariusz Kłopotowski, Maciej Dąbrowski, Natalia Kopyłowska-Kuć, Piotr Kołsut, Mariusz Kuśmierczyk

**Affiliations:** 1 Department of Cardiac Surgery and Transplantology, The Cardinal Stefan Wyszyński Institute of Cardiology, ul. Alpejska 42, 04-628 Warsaw, Poland; 2 Department of Congenital Cardiac Defects, The Cardinal Stefan Wyszyński Institute of Cardiology, Warsaw, Poland; 3 Department of Interventional Cardiology and Angiology, The Cardinal Stefan Wyszyński Institute of Cardiology, Warsaw, Poland; 4 Students’ Scientific Group at the Department of Cardiosurgery and Transplantology, The Cardinal Stefan Wyszyński Institute of Cardiology, Medical University of Warsaw, Warsaw, Poland

**Keywords:** Hypertrophic obstructive cardiomyopathy, HOCM, myectomy, mitral valve repair

## Abstract

**Background:**

Myectomy remains the standard surgical treatment of patients with hypertrophic cardiomyopathy (HOCM). New surgical methods developed in the last decades mainly address the mitral valve and are controversial because of their conflicting assumptions. This study assesses the influence of anterior mitral valve leaflet (AML) length and the anterior-posterior diameter of the mitral annulus (MAD) on dynamic left ventricle outflow tract obstruction and mitral regurgitation (MR) after extended myectomy.

**Methods:**

We retrospectively analysed the transthoracic echocardiograms (TTE) of 36 patients. AML length and MAD were obtained from TTE performed before the operation. The greatest maximal left ventricle outflow tract (LVOT) gradient and MR registered in follow-up were analysed. After surgery, patients were divided into two groups; those with moderate or milder MR and/or an LVOT gradient < 30 mmHg (responders), and those with more than moderate MR and/or an LVOT gradient ≥30 mmHg (non-responders).

**Results:**

Patients in responders group had significantly longer AML: 32.3 ± 2.3 mm vs 30.0 ± 3.8 mm (*p* = 0.03) [parasternal long axis view – PLAX view], 25.9 ± 2.3 mm vs 23.5 ± 2.7 mm (*p* = 0.008) [four chamber view - 4CH view] and larger anterior-posterior mitral annulus diameter 28.1 ± 2.8 mm vs 25.4 ± 3.2 mm (*p* = 0.011) than those in non-responders group. Among all analysed patients longer anterior mitral leaflet was correlated with lower postoperative LVOT gradient when measured in PLAX view (*p* = 0.02) and lower degree of MR due to systolic anterior motion (SAM) when measured in 4CH view (*p* = 0.009). Greater [AML x mitral annulus] ratio correlated with lower postoperative LVOT gradient in both projections: 4CH (*p* = 0.025), PLAX (*p* = 0.012). There was significant reduction in NYHA Class after surgery (*p* = 0.000). There were no significant differences in NYHA class after surgery (*p* = 0.633) neither in NYHA class reduction (*p* = 0.475) between patients divided into responders and non-responders group according to echocardiographic parameters.

**Conclusions:**

Patients with a longer AML and a greater diameter of the mitral annulus are less likely to have mitral regurgitation due to residual SAM and increased LVOT gradient after an extended myectomy. Division of patients according to echocardiographic criteria into responders and non-responders was not in concordance with clinical improvement.

**Trial registration:**

Retrospective study. Approved by ethics committee (IK-NPIA-0021-21/1763/19) at 16.01.2019.

## Background

According to recent European and American guidelines, surgical myectomy remains a gold standard in symptomatic patients with HOCM refractory to medical treatment. Sufficient extent of interventricular (IVS) shaving alleviates increased LVOT gradient and systolic anterior motion (SAM) [1]. Coexisted mitral valve leaflets and subvalvular apparatus pathology may contribute to suboptimal effect of isolated myectomy [2]. Nevertheless, additional procedures addressing elongated mitral leaflets or anteriorly displaced papillary muscle are required very rarely and there are no clear guidelines when to perform them. Decision whether to perform additional procedures on mitral valve may be particularly difficult in low- and middle-volume centres with limited experience in this field. Variety of surgical strategies, encompassing elongation as well as shortening of anterior mitral leaflet derives additional ambiguity in decision making. Mitral valve replacement is currently known as harmful and connected with inferior long-term prognosis, and numerous articles support this opinion [3, 4]. The mechanisms of dynamic LVOT obstruction are well known, it has been sufficiently proven that elongated AML contribute to dynamic increase of LVOT gradient among patients suffering from HCM [5, 6]. However, the conditions of blood flow after myectomy are different and the relationships between AML length, the anterior-posterior diameter of the mitral annulus, dynamic LVOT obstruction and MR were not investigated so far. The aim of our study was to find the correlation between AML length and the diameter of the mitral annulus measured preoperatively with echocardiographic markers of late surgical outcome – the greatest maximal LVOT gradient and MR, registered during follow-up.

## Methods

In the first step we investigated correlations in whole analysed population between the anterior mitral leaflet length, mitral annulus diameter (both measured preoperatively) and the greatest maximal LVOT gradient and the greatest mitral regurgitation registered in the follow-up. We calculated indices: [AML / annular diameter] and [AML x annular diameter] to find out how the combination of this two variables correlates with LVOT gradient and MR. In the second step the patients were divided into two groups; those with moderate or milder MR and/or an LVOT gradient < 30 mmHg (responders), and those with more than moderate MR and/or an LVOT gradient ≥30 mmHg (non-responders). These two groups were compared in terms of AML and MAD. The exclusion criteria were mitral valve regurgitation other than secondary to SAM, additional procedures on mitral leaflets or need for mitral or aortic valve replacement.

### Study population

We retrospectively analysed the echocardiograms and clinical data of 36 consecutive patients with HOCM operated in our institution from 2012 to 2016 (21 male patients, mean age 51 ± 14.77 years). The indication for surgery were persisting severe symptoms despite optimal pharmacological therapy: heart failure (NYHA III or IV) or syncope after exercise and LVOT gradient > 50 mmHg either with rest or with provocation. ICD (implantable cardioverter-defibrillator) was preoperatively implanted in 10 patients (27.8%). Previous alcohol septal ablation had been performed in 3 patients (8.3%). The maximal LVOT gradient before the myectomy was 96.53 ± 33.25 mmHg [Table 1]. Mitral regurgitation before the operation, judged as secondary to SAM was as follows: 1 severe, 19 moderate, 16 mild or none [Table 2]. Pharmacological therapy, comorbidities and distribution of NYHA Class are detailed in Tables 3, 4 and 5.

**Table 1:** Characteristics of study population – annulus diameter, AML 4 CH and AML PLAX were measured preoperatively

	Min–max	Average (SD)
Annulus diameter [mm]	21.0–34.0	27.1 (3.2)
AML 4CH [mm]	19.0–29.7	25.0 (2.7)
AML PLAX [mm]	22.3–36.0	31.4 (3.1)
The greatest gradient before operation [mmHg]	40.00–195	96.53 (33.25)
The greatest gradient after operation [mmHg]	5.00–110	28.86 (22.60)

SD: standard deviation, AML: anterior mitral leaflet, 4CH: apical four chamber view, PLAX: parasternal long axis view

**Table 2:** Mitral regurgitation due to SAM assessed in TTE: before surgery and the highest grade of MR measured in follow-up

	Before operation	After operation
None^a^	2 (5.6%)	7 (19.4%)
Mild^a^	14 (38.9%)	24 (66.7%)
Moderate^a^	19 (52.8%)	3 (8.3%)
Severe^a^	1 (2.8%)	2 (5.6%)

^a^In accordance with the 2017 guidelines of the American Society of Echocardiography

**Table 3:** Preoperative pharmacological therapy

Medicine	*N* (%)
Amlodipine	9 (25)
Verapamil	4 (11.1)
Beta blockers	34 (94.4)
ACEI/ARB	12 (33.3)
MCRA	4 (11.1)
Amiodarone	2 (5.6)
Diuretics	5 (13.9)

ACEI: angiotensin-converting-enzyme inhibitor, ARB: angiotensin II receptor blockers, MCRA: antimineralocorticoid

**Table 4:** Patient comorbidities

Comorbidity type	*N* (%)
Hypertension	12 (33.3)
CAD	2 (5.6)
CAD after PCI	5 (13.9)
Atrial fibrillation	14 (38.9)
Tobacco use	7 (19.4)
COPD	3 (8.3)
Hyperlipidaemia	16 (44.4)
Obesity	8 (22.2)
Diabetes mellitus	4 (11.1)
Chronic kidney disease	2 (5.6)
TIA/stroke	2 (5.6)
Sleep apnoea	1 (2.8)
Nonsustained ventricular tachycardia	6 (16.6)
Syncopes	22 (61.1)

CAD: coronary artery disease, PCI: percutaneous coronary intervention, COPD: chronic obstructive pulmonary disease, TIA: transient ischemic attack

**Table 5:** NYHA Class before and after surgery

	Before surgery	After surgery
NYHA I	0 (0%)	20 (55.6%)
NYHA II	8 (22.2%)	13 (36.1%)
NYHA III	26 (72.2%)	3 (8.3%)
NYHA IV	2 (5.6%)	0 (0%)

NYHA: New York Heart Association classification

### Echocardiographic assessment

**Fig. 1:**
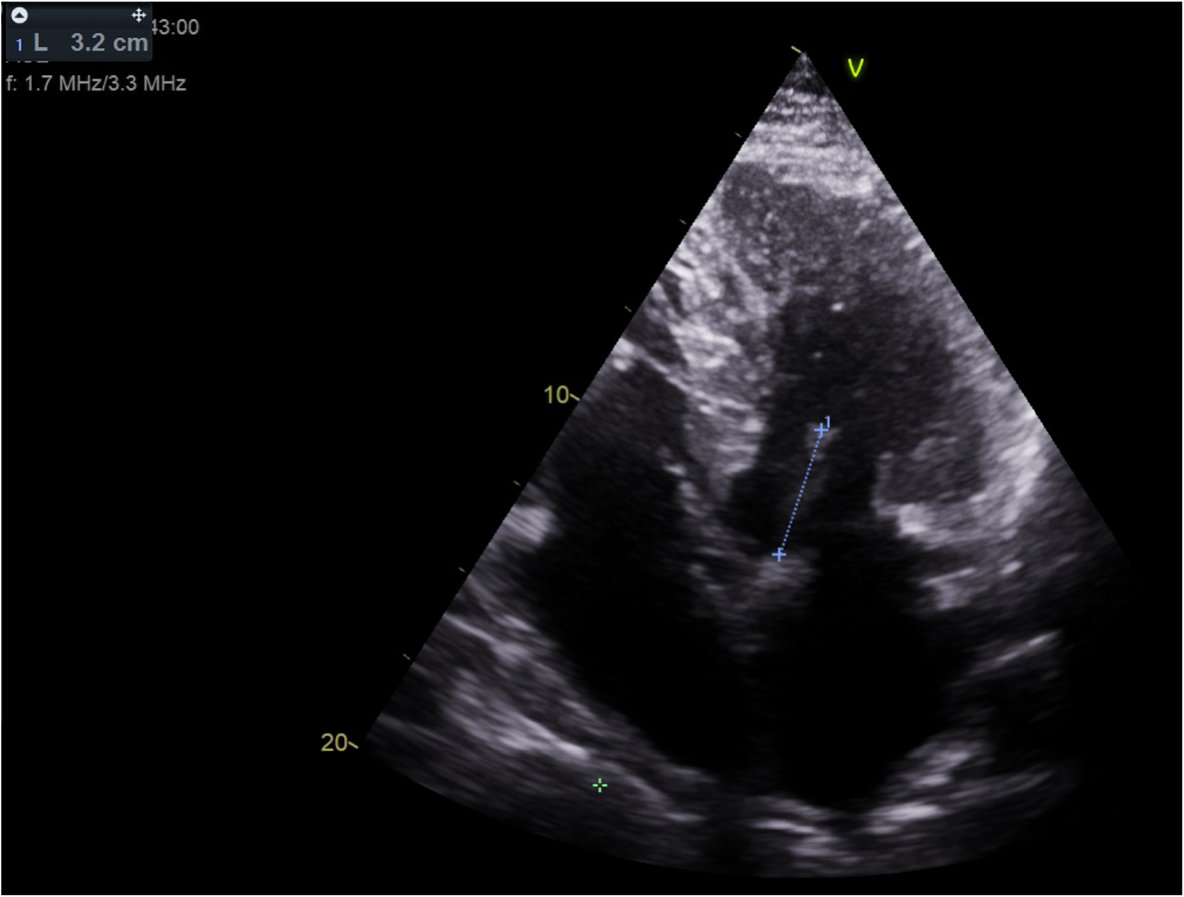
Anterior mitral valve leaflet length, apical four chamber view (4CH)

**Fig. 2:**
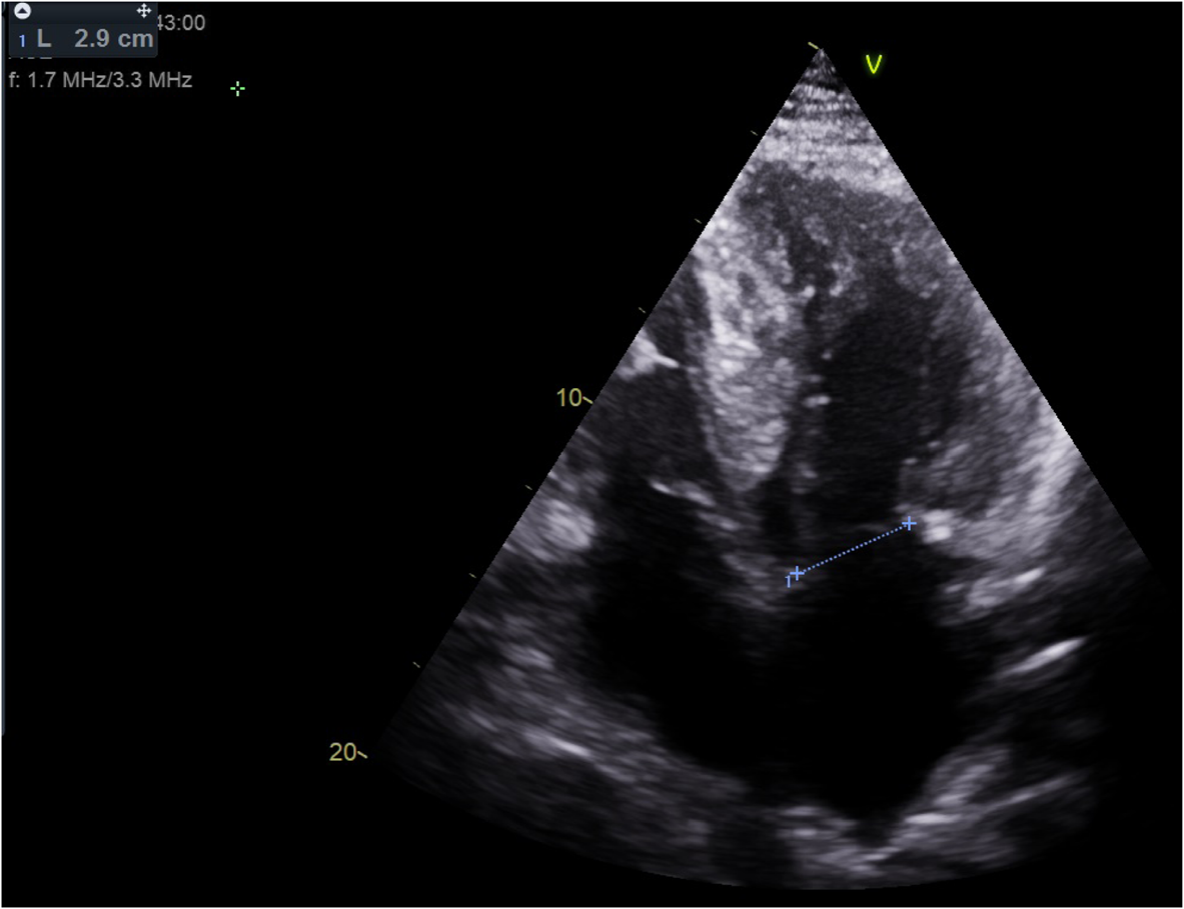
Anterior-posterior diameter of mitral annulus, apical four chamber view (4CH)

Anterior mitral leaflet length was measured at the A2 segment in two standard projections: the parasternal long axis three chamber view (PLAX) and the apical four chamber view (4CH) [Fig. 1] [Table 1]. The AML length was measured three times and the average was calculated for further analysis. The anterior-posterior diameter of the mitral annulus was measured in abovementioned projections and the average calculated for analysis [Fig. 2]. AML length and MAD were obtained from echocardiograms performed before operation. All available echocardiograms performed during follow-up were reviewed and the greatest registered LVOT gradient and MR being further analysed. Mitral insufficiency was classified as mild, moderate or severe in accordance with the 2017 guidelines of the American Society of Echocardiography. Echocardiographic assessments were made by cardiologist experienced in HCM and in the case of excentric multijet regurgitant jet, mitral regurgitation was assessed visually. Indices for each projection were calculated: [AML / annular diameter] and [AML x annular diameter] [Table 6].

**Table 6:** Calculated indices

	Min–max	Average (SD)
AML 4CH x annulus	4.56–9.86	6.78 (1.2)
AML PLAX x annulus	5.72–11.73	8.53 (1.45)
AML 4CH / annulus	0.60–1.21	0.93 (0.14)
AML PLAX / annulus	0.74–1.51	1.17 (0.16)

AML: anterior mitral leaflet, 4CH: apical four chamber view, PLAX: parasternal long axis view

### Surgical treatment

Procedures were performed via classic median sternotomy. After standard cannulation cold blood cardioplegia was administrated to the aortic root. Visualisation of the intracardiac structures was achieved via a transverse aortotomy. An extended myectomy was performed in each patient. Excision of IVS muscle was carried out from about 1.5 cm beyond the level of right coronary artery to the left lateral free wall in width and to the level of papillary muscle attachment in length. Both the mitral valve and the subvalvular apparatus were assessed intraoperatively. When localised intraoperatively, accessory chordae connected with the ventricle’s free wall were excised depending on the surgeon’s judgement. Mitral valve competence and LVOT gradient were assessed by TEE after weaning from cardiopulmonary bypass (CPB). All analysed patients have no more than mild MR and no more than 20 mmHg LVOT gradient immediate after weaning from CPB. Following concomitant procedures were one CABG, one aortic tube graft replacement and two ablations with left atrial appendage closure.

### Ethics

The present study was conducted in accordance with the Declaration of Helsinki and was approved by the Local Ethics Committee.

### Statistical analysis

IBM SPSS 25.0 software was used for statistical analysis. Continuous variables were reported as mean ± SD. The Shapiro-Wilk test was performed to determine whether a sample of values followed normal distribution. Intergroup comparisons were made by the Student’s t-test, to compare NYHA classes we used Chi^2^ test. The Pearson and Spearman correlation was used depending if normal distribution or not was confirmed. The Wilcoxon’s test for dependent samples was used to assess the significance of the changes in the preoperative-postoperative measurements for quantitative and ordinal variables. Categorical variables are expressed as absolute numbers and their frequencies in percentages. A *p*-value < 0.05 was considered significant.

## Results

**Fig. 3:**
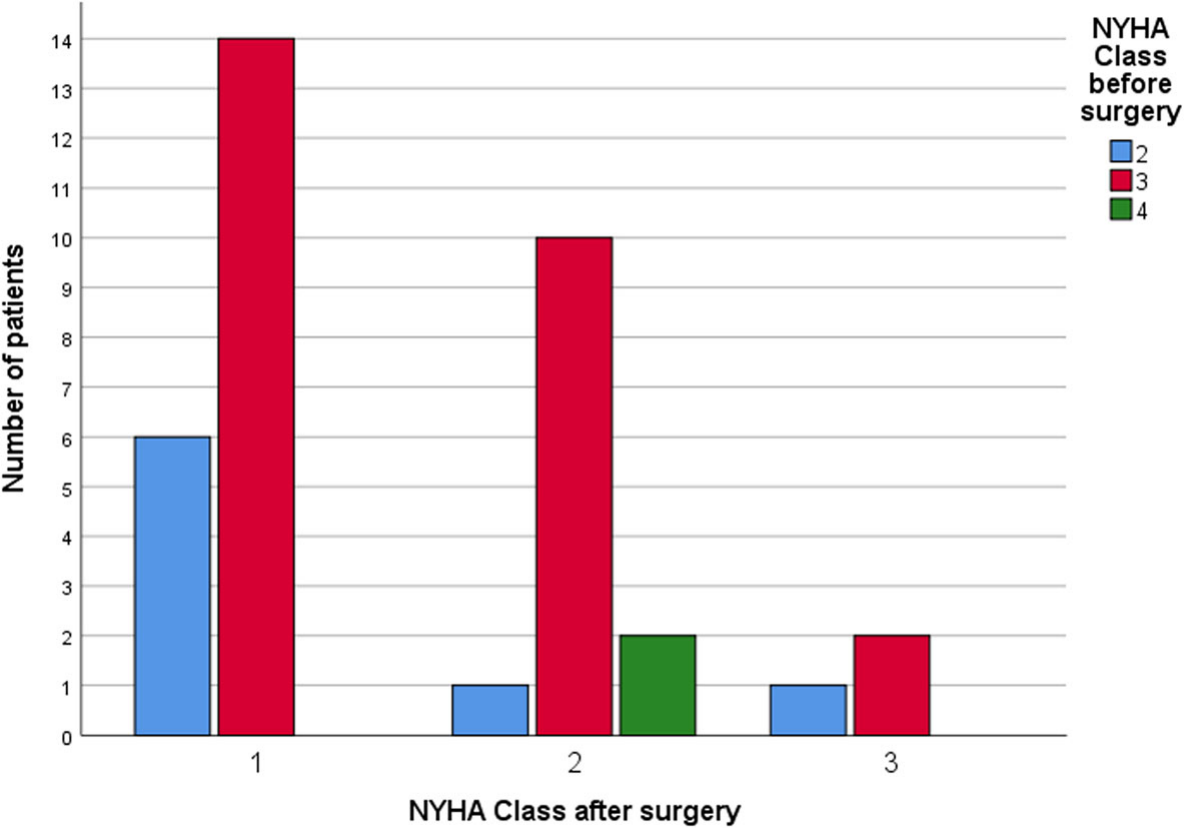
Changes of NYHA Class, before and after surgery

The mean follow-up time after the myectomy was 23.7 ± 18.9 months. We notated significant reduction in NYHA class after surgery (*p* = 0.000). Distribution of NYHA class before and after surgery is detailed in Table 5. Changes in NYHA classes before and after surgery are presented in Fig. 3. Two patients did not change their NYHA class III after surgery and there was progression from NYHA class II to III in one patient. These three patients were in echocardiographic responders group. The lack of clinical recovery was caused by serious comorbidities: COPD, rhythm disturbances and chronic postpericardiotomy syndrome treated with steroids. One patient was re-operated on in our centre due to recurrent high LVOT gradient (91 mmHg) due to SAM with mild MR and symptoms: syncopes provoked by effort and NYHA class II. There was small left ventricle cavity 3,7 cm, mitral valve leaflets were fibrotic with calcifications at the base of posterior mitral leaflet. In this case we performed re-do myectomy and decided to replace mitral valve. Another two patients in whom significant MR were measured have improved after modification of medical treatment and achievement of good rate control in atrial fibrillation. Followed echocardiograms revealed no more than moderate mitral insufficiency. Among all analysed patients we noted a significant reduction in the degree of MR after the operation (p-0.001). The greatest LVOT gradient in follow-up was 28.86 ± 22.6 mmHg, reduction in the LVOT gradient after operation was significant (p-0.001). The mean AML length was 2.5 ± 0.27 cm for 4CH view and 3.14 ± 0.31 cm for PLAX view. The mean anterior-posterior mitral annulus diameter was 2.71 ± 0.32 cm. Calculated indices are detailed in Table 6. Overall, longer AML (measured in 4CH view) correlated with lower degree of MR due to SAM (p-0.009) and lower LVOT gradient (p-0.02) (for AML measured in PLAX view) in follow-up. Higher [AML x annulus] ratio correlated with lower LVOT gradients after myectomy for AML measured in both projections: 4CH (p-0.025) and PLAX (p-0.012). Mitral annulus diameter has not had any impact neither on MR nor on LVOT gradient when correlated as isolated parameter in analysed population. Correlations with the LVOT gradient and MR after the operation are detailed in Table 7. After dividing our population into two groups: responders and non-responders we found-out that in responders group there were significantly more mail patients (*p* = 0.004). Responders have slightly but significantly longer AML measured in both projections: 32.3 ± 2.3 mm vs 30.0 ± 3.8 mm (p-0.03) [PLAX view], 25.9 ± 2.3 mm vs 23.5 ± 2.7 mm (p-0.008) [4CH view]. Anterior-posterior mitral annulus diameter was also larger in responders group 28.1 ± 2.8 mm vs 25.4 ± 3.2 mm (p-0.011). Patients in responders group have significantly larger [AML x annulus] ratio for AML measured in both projections: PLAX (p-0.002), 4CH (p-0.001). There were no significant differences in NYHA class before surgery (*p* = 0.229), after surgery (*p* = 0.633) neither in the degree of reduction of HYHA class (*p* = 0.475) among echocardiographic responders and non-responders group. The intergroup comparison is detailed in Table 8.

**Table 7:** Studied correlations with postoperative LVOT gradient and MR

	Gradient after operation *p*-value	MR after operation *p*-value
AML PLAX	(−) 0.020	(−) 0.329
AML 4CH	(−) 0.076	(−) 0.009
AML 4CH x annulus	(−) 0.025	(−) 0.846
AML PLAX x annulus	(−) 0.012	(+) 0.296
AML 4CH / annulus	(−) 0.842	(−) 0.596
AML PLAX / annulus	(−) 0.445	(+) 0.497
annulus	(−) 0.206	(+) 0.843

(−) negative correlation, (+) positive correlation

4CH: apical four chamber view, PLAX: parasternal long axis view

**Table 8:** Intergroup comparison using Student t-test, patients divided according to the echocardiographic outcome after surgery. NYHA class was compared using Chi^2^ test

	Responders *n* = 22	Non-responders *n* = 14	*p*-value
Gender (man/woman)	17/5	4/10	0.004
	Average (SD)		
Age [years]	48.68 (16.98)	55.43 (9.58)	0.137
AML 4CH [mm]	25.9 (2.3)	23.5 (2.7)	0.008
AML PLAX [mm]	32.3 (2.3)	30.0 (3.8)	0.030
Annulus [mm]	28.1 (2.8)	25.4 (3.2)	0.011
AML 4CH x annulus	7.07 (1.12)	5.93 (1.05)	0.011
AML PLAX x annulus	8.91 (1.33)	7.41 (1.27)	0.006
AML 4CH / annulus	0.94 (0.13)	0.93 (0.18)	0.92
AML PLAX / annulus	1.18 (0.14)	1.16 (0.22)	0.835
NYHA Class before surgery	–	–	0.229
NYHA Class after surgery	–	–	0.633
NYHA Class change before-after	–	–	0.475

SD: standard deviation, AML: anterior mitral leaflet, 4CH: apical four chamber view, PLAX: parasternal long axis view, NYHA: New York Heart Association classification

## Discussion

Extended myectomy first described by Messmer [7] and Shoendube [8] is a well proven method of treating patients with obstructive cardiomyopathy without concomitant structural mitral valve pathology. In most cases a myectomy alone sufficiently reduces the LVOT gradient, mitral valve insufficiency and partially or completely abolishes SAM. Mortality and life expectancy after the procedure is similar to the general population [9]. About 1–6% of patients require a re-do operation because of recurrent LVOT gradient and symptoms [10]. In our population one patient needed re-do operation because of high LVOT gradient and symptoms (2,8%). Patients with LVOT gradient equal 30 mmHg or more and MR greater than moderate did not differ significantly from those with lower gradients regarding to NYHA class. Patients who failed to improve their NYHA class had severe comorbidities (COPD, rhythm disturbances- nsVT and AF, postpericardiotomy syndrome) and it seemed that these were the causes of failed clinical improvement. Higher percentage of male patients in echocardiographic responders group has not been reported in literature so far. Midventricular obstruction and persistent SAM causing a dynamic increase in the gradient in LVOT are the main reasons reported as responsible for failed procedures [10] what is in concordance with our experience. Deeper resection of the IVS muscle has been proven to sufficiently reduce the gradient in re-do patients [10]. In our re-do case we decided to replace mitral valve because it was moderately degenerated and we were warried of persistent LVOT gradient due to SAM. Coexisted pathology of the mitral valve leaflets and subvalvular apparatus like elongation of the leaflets, anterior displacement of the papillary muscles and accessory chordae are in last decades more and more often accused of being the reason of inferior surgical outcome in some selected cases [11]. In our previously reported results, mostly incomplete SAM was noted in 12% of patients operated on [12]. Thus forced surgeons to develop surgical techniques addressing abovementioned pathologies. The most widespread and accepted methods are mobilisation of the papillary muscles and resection of the secondary chordae which pulls the AML towards the outflow tract [13, 14]. More controversial are techniques involving the anterior mitral valve leaflet. Theoretically, longitudinal or perpendicular plication of the AML may stiffen the mid portion of the leaflet, preventing its billowing and shortening the AML [15–19]. The most common complication is mitral valve insufficiency caused by the procedure itself [20]. The rationale for leaflet extension is the assumption that the AML will be pushed away from the LVOT like a parachute [21–23]. Objective parameters that could suggest which technique would be more suitable to diagnosed pathology are lacking and the surgical approach used by each HCM centre is mostly based on its experience. The only suggestion that AML shortening may be beneficial is the proof that increased an AML/LVOT ratio is associated with a recurrent increased LVOT gradient [11]. The aim of our study was to find if the AML length and the mitral valve annulus may be responsible for increased LVOT gradient and mitral regurgitation due to SAM in of follow-up. The least favorable measurement of LVOT gradient and mitral regurgitation reflects more accurately real-live conditions. It has been already proven that elongated AML cause dynamic increase of LVOT gradient in HOCM patients before surgery [5, 6]. After surgical ameliorating of LVOT flow pattern in left ventricle changes [24] and hemodynamic conditions are incomparable. We proved that after myectomy patients benefit from longer AML. Additionally, larger mitral annulus combined with AML contribute to beneficial effect of myectomy.

### Limitations

Study was performed in a single centre and analysed TTEs were performed by various cardiologists on small sample size consisted of 36 patients. Patients with additional procedures on mitral valve which often had untypical pattern of hypertrophy were excluded from the study. Operated patients were referred to our institution from all regions of Poland and therefore some of them were followed by local clinicians after standard one month visit.

## Conclusions

With this retrospective study, we proved that patients with a longer AML and a greater anterior-posterior diameter of the mitral annulus are less likely to have mitral regurgitation due to residual SAM and increased LVOT gradient after a myectomy. Division of patients according to echocardiographic criteria into responders and non-responders was not in concordance with clinical improvement. Studies on larger samples need to be performed to objectively justify additional procedures on mitral valve.

## Data Availability

The datasets used and analyzed during this study are available from the corresponding author on reasonable request.

## Abbreviations

4CH view: four chamber view,
AF: atrial fibrillation,
AML: anterior mitral valve leaflet,
CPB: cardiopulmonary bypass,
HOCM: hypertrophic cardiomyopathy,
ICD: implantable cardioverter-defibrillator,
IVS: interventricular shaving,
LVOT: left ventricle outflow tract,
MAD: mitral annulus diameter,
MCRA: antimineralocorticoid,
MR: mitral regurgitation,
nsVT: nonsustained ventricular tachycardia,
NYHA: New York Heart Association classification,
PLAX view: parasternal long axis view,
SAM: systolic anterior motion,
SD: standard deviation,
TEE: transesophageal echocardiogram,
TTE: transthoracic echocardiogram
